# “They have their own people”: Emotional connections, community belonging, and Chinese gay, bisexual and other men who have sex with men (GBMSM) college students’ needs for sexual health support

**DOI:** 10.1371/journal.pone.0291550

**Published:** 2023-09-20

**Authors:** Minhui Yang, Chunyan Li, Kaiyue Zou, Yun Liang, Rudong Zhang, Kun Tang

**Affiliations:** 1 Department of Social and Behavioural Health Sciences, Dalla Lana School of Public Health, University of Toronto, Toronto, Ontario, Canada; 2 Tokyo College, University of Tokyo, Tokyo, Japan; 3 Department of Health Behavior, Gillings School of Global Public Health, University of North Carolina, Chapel Hill, NC, United States of America; 4 Epidemiology Department, Bloomberg School of Public Health, Johns Hopkins University, Baltimore, MD, United States of America; 5 Vanke School of Public Health, Tsinghua University, Beijing, China; Torrens University Australia, AUSTRALIA

## Abstract

Gay, bisexual and other men who have sex with men (GBMSM) college students in China have unique sexual health challenges, including a higher risk of HIV infection, stigma and discrimination against LGBTQ (lesbian, gay, bisexual, transgender, and queer) population, and limited access to LGBTQ-affirmative sexual health support. Nonetheless, previous research or policymaking has rarely addressed the students’ needs for sexual health support from their perspectives. This study aims to explore GBMSM college students’ perceptions and attitudes to current sexual health resources, the challenges they encounter, and their expectations to acquiring LGBTQ-affirmative sexual health information and services. The exploration was carried out through field visits and in-depth interviews with 26 GBMSM college students and eight relevant stakeholders in five cities in China. Qualitative thematic analysis was applied to the interview transcripts and fieldwork memos. Four themes emerged around the preference and needs of GBMSM students in dealing with their sexual health challenges: the association between tackling sexual health challenges and entering LGBTQ communities, the roles of emotional attachment in shaping the preference for HIV-related care and support, the desired modes of acquiring sexual health support, and the current unmet service needs. We discovered that the information-and-care-seeking behaviors of GBMSM college students are highly influenced by and connected to their participation in online and in-person LGBTQ communities. Due to the overall stigmatizing sociocultural environment of LGBTQ in China, GBMSM college students tend to rely on LGBTQ communities, seeking trust and a sense of belongingness for tackling their sexual health challenges. Conventional school-based sexual health educational programs, which often apply top-down, stigma-and-fear-based, and non-LGBTQ-inclusive teaching strategies, rarely help GBMSM college students to solve sexual health problems in real life. GBMSM college students are eager to have LGBTQ-affirmative "health managers" who can understand their emotional experiences and interpersonal contexts and assist them with sexual health issues. However, such support is generally perceived as limited by the students. Our study highlights these unmet needs of the GBMSM students and emphasizes the importance of developing future LGBTQ-affirmative sexual health programs among Chinese GBMSM college students and young GBMSM in general.

## Introduction

China’s gay, bisexual, and other men who have sex with men (GBMSM) have been disproportionately affected by the HIV epidemic with an increasing trend of new HIV infections in the past decade [1]. GBMSM account for over 80% of new HIV infections in major Chinese cities such as Beijing and Shanghai [1], the majority of whom are diagnosed with HIV in their 20s to 50s year old [2]. Several sociocultural factors were reported to be associated with higher HIV infection risk among GBMSM particularly young GBMSM. These factors include the wide spread of discrimination against HIV and non-heterosexuality in the general Chinese society [3], suboptimal social and sexual health support [2,4,5], and a lack of comprehensive understanding about HIV and HIV prevention [6]. Faced with a repressive social environment, engagement in unprotected sex behaviors is not an uncommon stress coping strategy among Chinese GBMSM who have experienced external discriminative reactions and internalized stigmatizations [3,5,6]. Previous studies in China indicated that Chinese GBMSM tended to conceal their sexual orientation by engaging in heterosexual marriages and avoiding participating in HIV testing and prevention programs in fear of unintended identity disclosure [4,6].

HIV and sexual health education for young GBMSM including college students have been proven effective in harnessing HIV transmission worldwide. However, GBMSM college students face multifaceted systemic barriers that hinder the effectiveness of such education in China. These barriers include the heterosexual-dominated sex education curriculum and the disconnection between schools and community-based organizations. Currently, mainstream school-based sex education in China primarily focuses on heterosexual relationships, failing to provide sufficient introduction to gender and sexuality diversity or how to manage non-heterosexual relationships [2,7]. The unique sexual health challenges and needs among gender and sexuality minority students, including GBMSM, such as psychological distress, and perceived and experienced stigma and discrimination against their sexuality, are often overlooked [8,9]. Although GBMSM have been recognized as a key population in Chinese government’s HIV prevention strategies since 2000s [10,11], and HIV testing programs have been expanded in collaboration with community-based organizations (CBO) [10,12] to reach the general public, such CBO-based HIV prevention programs are not commonly seen on campus or in collaboration with schools due to lack of administrative support from school leadership. Furthermore, the voices of GBMSM college students themselves are missing from the strategies for designing and implementing HIV prevention interventions.

In the absence of effective and LGBTQ-friendly sexual health education and support, many GBMSM students actively selected peer and online resources, such as LGBTQ-led CBO and LGBTQ influencers for relevant information and counselling, specifically for sexual health support [6]. Both LGBTQ-led CBO and LGBTQ influencers created an environment that fosters feelings of connectedness and belonging for the GBMSM college students [13]. Such connectedness has been proven significantly associated with the well-being of LGBTQ and young people. For instance, in the United States, black sexual minority men identified LGBT-affirming churches as sources of acceptance and emotional healing [14]. In Indonesia, GBMSM social networks functioned as a channel for local GBMSM to escape from homophobic cultural and social norms [15]. Similarly, in Uganda, micro-social environments were observed to “have the potential to exacerbate or reduce harm through shaping perceptions of health risks and practices”, including their behaviors related to receiving HIV tests and undertaking HIV prevention measures [16] (p.2192).

Our study contributes qualitative evidence to our current understanding of the needs and preferences of Chinese GBMSM college students for sexual health support. We analyze the sexual health challenges faced by GBMSM students and explore how they were affected by the interpersonal contexts and emotional connectedness enacted by LGBTQ communities. Recognizing the active subjectivities of the students, we aim to carry out our exploration from their standpoints and situated within their living experiences. We argue that the emotional and relational aspects play an essential role in the GBMSM students’ acceptance and preferences for sexual health support sources that inherit different priorities and values. Additionally, based on their own imagination and desires, we propose suggestions for future policymaking and intervention projects that could better support their sexual health wellbeing.

## Research methods

From June to December 2019, the authors MY and CL conducted in-person in-depth interviews with 26 GBMSM college students and eight stakeholders (officers of local CDC, staff of community-based organizations, and college and university faculties who were engaged in HIV prevention and sexual education. Field visits were made to five cities of the People’s Republic of China (hereafter, China): Beijing, Tianjin, Nanjing, Chongqing, and Kunming. GBMSM college students were eligible if they were 18 years old or above, Chinese citizens, enrolled in a college or university within China at the time of the interview, and capable of making voluntary decisions about whether to receive the interview. Stakeholder participants were included if they were 18 years old or above, Chinese citizens, working in or having working experience in HIV- and /or sexual-education-related organizations (such as government agencies, colleges, non-governmental organizations, and community-based organizations and groups), and capable of make voluntary decisions about participation.

GBMSM college student participants were primarily recruited through study post on social media platforms and online forums as well as follow-up snowballing referrals. The online recruitment was an open process and was only limited by the sample size and inclusion criteria. The stakeholders were recruited through convenient sampling and snowballing processes with support from national and local family planning associations, CDC, and community-based organizations. Oral or written informed consent of the participants was obtained before the interviews were conducted depending on the participants’ choices. During and after the interviews, no participants dropped out or withdrew their consents of participation.

The interviews with both GBMSM college students and stakeholders were conducted by authors MY and CL, who were cis-gender female public health researchers with extensive experience working with Chinese GBMSM through conducting their master’s and PhD study projects. Neither MY or CL had had contacts with any research participants prior to the interviews. The interview questions touched upon topics including individual experience of sexual health education at college, self-identified sexual orientation, perceived social support for LGBTQ young people, sexual behavioral patterns, and HIV and other sexually transmitted infections (STIs) prevention knowledge, attitudes, and HIV-related prevention healthcare utilization. For interviews with key stakeholders, the questions included their working experience in the HIV/STI field and perceived barriers to promoting HIV prevention in China (particularly towards college students and young people). All interviews were conducted in a one-to-one (CL or MY as solo interviewer) or two-on-one (CL and MY as co-interviewers), private environment to ensure privacy and confidentiality. All participants were assured in the informed consent process that their identity would be confidential and they were encouraged to share their honest responses.

The duration of the interviews varied from 45 minutes to 90 minutes. The interviews were conducted, transcribed, and analyzed in Mandarin Chinese. Throughout and after the interviews, MY and CL kept field notes on their reflections about the interviews, observations on the local sites related to HIV prevention and sexual health of GBMSM college students, and met regularly to discuss overall research insights and data saturation. Participant recruitment and interviews were concluded once data saturation had been reached. Data saturation refers to the point at which interviews no longer provided new information or additional data pertaining to the themes of the interview. To maintain confidentiality, all identifiable information of the interview participants was removed from the transcriptions and notes during the fieldnote writing and transcribing processes.

The analysis of interview transcriptions and field notes was collaboratively conducted by authors MY, CL, and KZ using Dedoose (v. 8.0). An inductive thematic analysis was applied to data analysis [17]. The analysis began with reading and familiarizing the transcriptions of the interviews. During their first round of reading, the authors (MY, CL, and KZ) proposed and discussed initial themes and codes around HIV-related and sexual health services. Building upon the initial codes and themes, a tentative codebook was developed. Then, the three members coded the transcriptions and notes and wrote memos based on the initial codes. The codebook was iteratively reviewed and adjusted during each team meeting (with MY, CL, KZ, YL, and KT) to ensure the themes are streamlined and organized. Drawing on the outstanding codes and themes of the codebook, MY and CL developed an outline of a manuscript on the current circumstances and unmet needs of sexual health support of the GBMSM college students. The parent codes of the codebook include social support (7 codes), identity (3 codes), dating (3 codes), sexual health education (7 codes), HIV testing (5 codes), discrimination and disclosure (10 codes).

The major components of the study, developing interview guide, recruiting participants, interviewing participants, and writing field notes were conducted by CL and MY. During the data collection, CL, a Chinese cis-gender woman, was a Ph.D. candidate in Health Behavior with over five years of research experience in HIV, GBMSM health and sexual health in China-based settings. MY also a Chinese cis-gender woman, was a critical qualitative researcher with experience in community-participatory research collaborating with international and community-based organizations engaging in HIV prevention among Chinese GBMSM population.

The study was approved by the Institution Review Board of Tsinghua University (Project number: 20190026).

## Results

**Fig 1:**
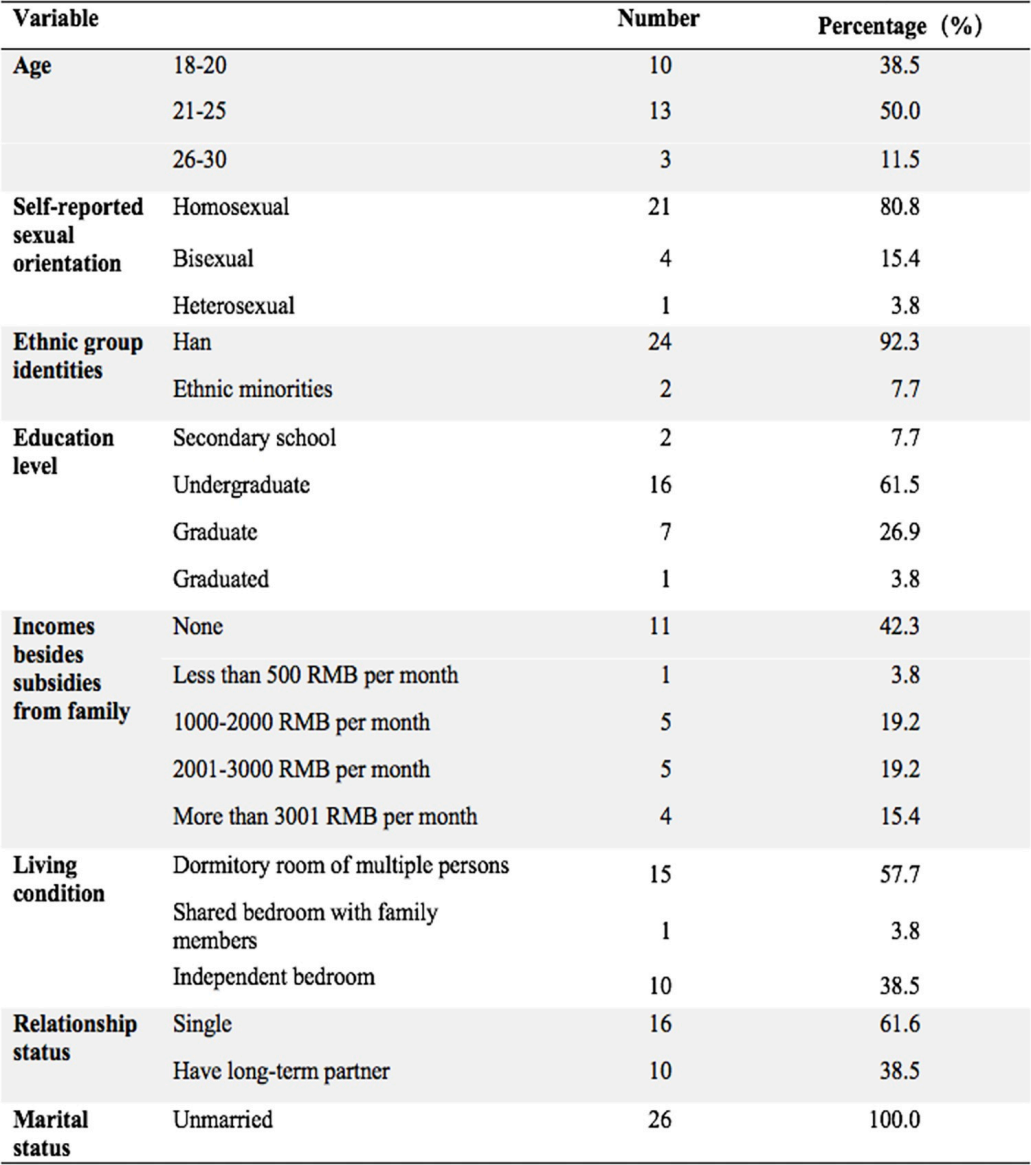
Demographic information of GBMSM college student participants.

The mean age of the GBMSM college student participants was 21.7 (range from 19 to 29, median = 21, standard deviation = 2.2). All participants self-identified as male and reported having had sexual activities with male partners. Among them, 24 had had anal sexual intercourse with males, and one only had non-anal-penetrative sexual activities. Twenty-one GBMSM self-identified as gay, four as bisexual, and one as heterosexual. More detailed demographic information is shown in Fig 1.

Overall, the GBMSM college students expressed an urgent need for more LGBTQ-affirmative social support and counseling, and identified the current gaps of such support in school-based sexuality education curriculum. LGBTQ communities or influencers were commonly mentioned by GBMSM students as major sexual health information sources. They include peer leaders, social media influencers, and online social groups. GBMSM college students were eager to have health professionals in LGBTQ communities who could assist them in tackling specific HIV-related and other sexual health problems, especially with emerging prevention methods such as Pre-Exposure Prophylaxis (PrEP). Even if the students were equipped with HIV prevention and sexual health knowledge, they expressed the need for personal counselling–preferably with someone coming from the same community—when they were supposed to apply acquired knowledge to deal with particular sexual health challenges. Furthermore, despite the students’ needs for on-campus sexual health support, what school-based intervention programs provided was insufficient and sometimes even had the opposite effects due to the current shame-based and fear-based approaches. The fear-based and shame-based education further stigmatized same-sex sexual behaviors, stimulated resistant emotions among the students, and decreased the students’ willingness to receive school-based HIV prevention and sexual health education, even if available.

### Explorations of sexualities, LGBTQ communities, and sexual health

We found that the GBMSM college students’ exposure to sexual health information was associated with their explorations of sexualities. Overall, most of the participants had initiated their explorations of sexual orientation before joining our interviews. Many of them reflected that their explorations started in high school, as they had experienced feelings of curiosity and interest in male classmates and celebrities. The explorations were described as entanglements and iterations of roughly five processes: self-motivated information seeking, browsing shows and literature related to LGBTQ culture, joining local and online LGBTQ communities, communicating with close peers, and having sexual activities with others. Since there are stigma and discrimination against sexual and gender minorities in mainstream Chinese society, the students tended to explore and socialize in online spaces such as social media platforms and forums, except for limited numbers of in-person spaces hosted by LGBTQ-friendly organizations. As they reflected, sexual health had been an essential topic of communication and interactions in these online and in-person contexts of exploring sexualities.

China-based online spaces provided by communication and social network sites such as Weibo, Baidu Tieba, and WeChat, video sharing sites such as Bilibili, and some online forums were the most common choices of the GBMSM college students when they were looking online for communities and peers. Peer leaders and social media influencers who were active in the mentioned online spaces attracted certain groups of people by sharing their thoughts and knowledge of sexuality and sexual health.

“*P*: *I think the internet is a very important channel*. *No matter for our generation or people younger than us*, *we get to know about (sexual health) through the internet*. *There are some movies*, *videos*, *and scientific lectures*. *For example*, *there are some accounts on Bilibili and also accounts on WeChat*, *like the one of [dating app name] and others*.*” (Participant_06*, *a GBMSM college student)*

“*I*: *When you browse things online*, *which websites would you use*?

*P: Tieba and Weibo. And, now, also Bilibili. There are some vloggers’ works on Bilibili that explain this thing to you. There are also videos, which are less boring than the school’s online courses.” (Participant_22, a GBMSM college student)*

Networks of mutual help and mutual education emerged around the accounts of peer leaders and social media influencers. Followers engaged with daily feeds and participated in discussions with other followers via comment, repost, and group chat functions. Unlike unidirectional or often top-down teaching activities, this online education in the do-it-yourself style fostered extensive interactions among the followers of the influencers and in-depth participation of the members of online forums and chat groups.

“*I*: *How would you usually acquire information from online channels*?

*P: I would read the contents posted by the accounts that I subscribed to on WeChat. For example, [dating app name] is operating its account on WeChat, and they have this kind of stuff. And, there are some accounts operated by gay individuals, and they would post things to meet their subscribers’ needs…” (Participant_12, a GBMSM college student)*

Meanwhile, the GBMSM college students were aware that peer leaders and social media influencers might not be able to provide the most professional and accurate health information. Therefore, if necessary, they would do further research with more academic and professional resources. Communication on social platforms served as an opportunity for the peer-to-peer exchange of sexual health information. The formation of LGBTQ communities and peer support for sexual health were mutually encouraged in online spaces.

GBMSM students also utilized in-person community-based peer support as a means of acquiring sexual health knowledge and seeking acceptance and affirmation of their sexualities. LGBTQ-led local CBO served as social hubs of local sexual and gender minorities, providing HIV prevention services. Additionally, public health authorities intentionally selected existing social hubs to undertake HIV prevention interventions because of their outreaches to the LGBTQ population. In both cases, the HIV prevention interventions served the dual purpose of education and socialization. GBMSM college students shared that they were initially introduced by friends to sexual-health-related activities held by local LGBTQ-led CBO when they disclosed their sexual orientations and mentioned their desire to mingle with “similar people”. Through participating in educational activities and receiving HIV counselling and testing, some students managed to become involved in local LGBTQ communities and form a long-term collaborative relationship with the LGBTQ-led CBO. The students reflected that they would turn to the organizations not only for sexual health information but also, more essentially, for the organizations’ staff whom they deemed trusted helpers in dealing with troubles related to sex and sexual health.

*The anticipated community-based “health managers”*

In the face of HIV transmission risk and other sexual health challenges, GBMSM college students chose trusted personalized (but also professional) support over standardized education curricula. As described above, many GBMSM college students were able to access sexual health information by themselves via online social and professional channels. Therefore, top-down educational programs were considered less desirable than personalized information and counselling services that would help the students tackle emerging sexual health challenges. Although it did not exist in their living contexts, the latter role was named and anticipated by some of the students as a “health manager”.

“*I think the dissemination of knowledge has been done enough*, *and people are all following some accounts on WeChat*. *But*, *this kind of one-on-one personalized (issues)*, *and some information that is not easy to find online… One may have some special incidents and need help*, *and they don’t know from where they could get it*.*” (Participant_17*, *a GBMSM college student)*

In the GBMSM college students’ conceptualization, a “health manager” would be (1) someone who could help them get instant access to healthcare providers for HIV testing and post-exposure prophylaxis if they had high-risk sexual behaviors (participant_12); (2) someone who could provide them with or refer them to social work and mental health support for sexual and gender minorities (participant_07); (3) someone who could maintain online group chats for updating information on HIV and other STIs prevention and treatment (participant_07); and (4) someone who could host mutual help peer groups of sexual and gender minorities for their sexual and mental health issues (participant_08). During our field visits for this study, we found that although anticipated by many of the GBMSM interview participants, such health management and community building features had yet to be fully realized by any healthcare or educational stakeholders.

Interestingly, the services that existed and were assimilated with the anticipated duties of a “health manager”, having the attributes of being personalized, professional, and accessible and responding to GBMSM’s own health challenges, were given as a byproduct of HIV prevention programs of some LGBTQ-led CBO. During the study, we visited a city sexual health support for GBMSM college students was provided by a local LGBTQ-led CBO in the forms of HIV voluntary counselling and testing (VCT) service. These initiatives were historically sponsored by the local CDC and some international organizations under the national HIV prevention regimes. While supporting GBMSM in addressing encountered sexual health troubles were not the priority of these regimes, the CBO staff, to some extent, took on this responsibility alongside their assigned tasks, which primarily revolved around scaling up HIV testing.

### Positive feelings toward community-based LGBTQ-friendly services

Another important reasons that GBMSM college students mentioned that their preference of choosing CBOs for sexual health care was its accessibility. For the students, although they could receive, for example, HIV testing and counselling from local CDC and hospitals, the limited hours of operation of these providers often kept them away. In contrast, the staff of the CBO were usually personally connected with the GBMSM college students and could sometimes help with their immediate problems with sexual health even outside of their office hours. Moreover, the local CBO had several of their offices near university campuses such that the students could visit them conveniently, unlike the CDC and designated hospitals for HIV, the locations of which were not as physically accessible.

The GBMSM college students’ preference for community-based options was not only associated with the quality and accessibility of service but also greatly affected by factors in emotional and interpersonal domains. In addition to achieving HIV control goals, community-based HIV prevention programs have also incubated a local community of LGBTQ attending to not only their sexual health needs but also their social and emotional connections. Based on the connections, trust, and dependence emerged among the community members, including both CBO staff and their student clients. One of the students that we interviewed said:

“*So*, *once you know it (the community-based organization) is here*, *you don’t need to think about other choices*. *You would feel like it is enough to have them (the community-based organization’s staff)*.*” (Participant_22*, *a GBMSM college student)*

Senses of acceptance and belonging associated with shared experiences of being LGBTQ were narrated by local GBMSM students and CBO staff. As one of the staff (Participant_CS20) said, the local GBMSM knew that they were “*their own people*, *so they would trust the organization*”. For GBMSM college students, the organization was a reliable source of support, with which they seldom experienced being patronized and scrutinized but often felt cared and supported by peers. Moreover, since LGBTQ were still highly stigmatized and discriminated against by mainstream Chinese sociocultural, discussing and working with “*their own people*” to tackle sexual health issues also gave the students feelings of trust, privacy, and safety.

### Negative reactions to school-based fear-based education

Unlike the comrade-caring-infused support provided by local LGBTQ-led CBO, sexual health interventions at the universities, mainly in the form of educational materials and lectures, largely appeared to have limited influence among GBMSM college students. The students reported two main reasons contributing to this result. First, the school-based sexual health programs rarely attended to the specific challenges of the students. Second, the school-based programs tended to be deficient in building positive emotional connections with the students, and some even brought negative feelings such as fear and guilt.

Overall, there was a lack of school-based LGBTQ-friendly HIV prevention and sexual health resources in the cities we visited. Many of the universities where our GBMSM interviewees were attending lacked established sexual health education curricula. HIV prevention and sexual health programs were often carried out through one-time lectures and activities, which could cover only a small proportion of students. Compared to the general college student population, GBMSM college students were even less targeted, as they were less likely to join the lectures due to their marginalized social positions, fear of their sexual orientation being exposed, and lack of satisfaction with the usually heterosexual-oriented education contents.

In some universities, sexual health education was set up as an elective course either online or in-person. The GBMSM college students reflected positively on the courses as they could consolidate some basic knowledge on sexual health, while there was no component tailored to LGBTQ population and the students’ unique health challenges in any one of the courses. GBMSM college students expressed a double-paradoxical attitude about whether the sexual health of LGBTQ should be incorporated into school-based curricula. On the one hand, they anticipated getting more LGBTQ-specific health information from school-based courses, a source that they deemed as reliable for its accuracy. However, they also found that sexual health education that targeted the general population of students could hardly cover all topics concerning LGBTQ population. On the other hand, despite their desire to have a course particular for the LGBTQ population, they also feared that participating or expressing interest in such a course would automatically expose their sexual orientations to the university authorities due to anticipated discrimination. These contradictory concerns of the students indicate the lack of trust and connections between the students and the universities’ educators and authorities. Although the students realized that their universities could be an agency for providing them with sexual health resources, they did not receive sufficient acceptance and respect from the educators that would convince them to rely on the school-based programs and use the resources without concerns.

Among the existing school-based sexual health programs, fear-based and shame-based interventions further deterred the emotional connections between the authorities and GBMSM college students. It prevented the students from trusting the universities to provide LGBTQ-friendly sexual health support. For example, the CDC of one city initiated a program to put posters in public men’s bathrooms on campus, written as “*there are 7 men-who-have-sex-with-men in every 10 HIV-positive cases*” (partcipant_22, a GBMSM college student). This homophobic-implied narrative deepened the stereotypical mis-association of male same-sex sexual behaviors and HIV transmission and the stigmatization of GBMSM. As participant_22 said, *“many straight men and women would think that this is a gay disease*, *and it is none of their business*. *So*, *if you intentionally pursue information on this thing (HIV)*, *people would look at you differently (and think that you are gay)*.*”* The fear-based and shame-based interventions made the GBMSM feel that they had to disguise their sexualities and avoid interactions with the university and public health authorities to protect them from being identified and discriminated against.

## Discussion and limitations

Through qualitative in-depth interviews with GBMSM college students and relevant stakeholders, we explored the perceived barriers, facilitators, and unmet needs of the GBMSM college students regarding sexual health challenges. The study specifically focuses on collecting and analyzing the students’ own narratives on their experience of acquiring sexual health information and services. Our interviews revealed informative and reflective agencies demonstrated by GBMSM college students as they actively take care of their own sexual health. The strong sense of self-care and self-optimization exhibited by GBMSM college students guided us to approach their living experiences of sexual health from their own standpoint.

We discovered that GBMSM college students’ exposures to sexual health knowledge and information were closely associated with their explorations of sexual orientations, and both of the processes were embedded in their interpersonal relationships with online and in-person LGBTQ communities. For the GBMSM college students, knowledge and information have become accessible through open online resources and peer networks; while the need for instant care and support facing emerging sexual health troubles, such as unprotected sexual intercourse, were still unmet. Emotional connections and interpersonal relationships played essential roles in determining the support-seeking behaviors of the GBMSM college students. Facing the homophobic mainstream social environment of China, the providers of such care and support were preferably their peers within LGBTQ communities, to whom they feel trust and a sense of belonging. This finding is aligned with results of other studies in various sociocultural settings. A cross-sectional study involving participants from 145 countries revealed that engagement with gay communities and connections with service providers is a facilitator to GBMSM to get HIV-related services [18]. A more recent qualitative study conducted in Indonesia particularly addresses social support as pivotal in GBMSM’s decision on participating in HIV testing and active condom-using [19].

In recent years, the proliferation of social media and online forums has provided LGBTQ individuals in China with a new platform to connect and interact with their communities [13]. The GBMSM college students find the anonymity of online spaces safer and more comfortable, as they can avoid confronting and being intruded on by the homophobic mainstream sociocultural environment of China. Online spaces have gradually become an essential channel for the GBMSM students to actively delve into sexuality and sexual health information [6]. In addition to online sources, LGBTQ-led CBOs that provide HIV prevention programs to the GBMSM population are another important resource relied upon by students for information, services, and care. Within these online and in-person LGBTQ communities, the students experience a sense of belonging, attentiveness, and trust that motivates them to choose community-based resources when facing emerging sexual health challenges.

Compared to online channels and community-based organizations, school-based programs provided by the authorities of universities and public health sectors appear to be less utilized by the GBMSM college students. Although some of the students find these authoritative sources of information and services more reliable, they are deemed as more difficult to access, less attending to the unique health challenges of LGBTQ, and less connected to the emotions and living experiences of the students. Moreover, the fear-based and shame-based approaches to sexual health education implemented by universities and public health authorities further repel the students and undermine their hopes of receiving inclusive and LGBTQ-friendly support. The fear-based and shame-based approaches may also intensify the stigmatization and discrimination of sexual and gender minorities [20], which has nevertheless been proven to be one of the major risk factors for HIV transmission and other sexually transmitted infections among GBMSM in China [21,22].

Our study shows new landscapes of sexual health care of GBMSM college students in terms of their self-perceived capacity and channels of acquiring knowledge and information. First, with access to sources and peers enabled by online spaces, young GBMSM feel more capable of and become more active in exploring the topics of their own concerns. Despite the students’ worries about their reliability, online sources are used extensively by them for sexual health information and knowledge. Correspondingly, the acceptance of conventional ways of disseminating knowledge, school-based teaching, may further diminish among GBMSM college students. Second, the inclusiveness and anonymity of online spaces determine that their users can no longer be categorized and intervened separately based on their ages and educational levels. Many of our interview participants reflected that they had already searched for sexualities and sexual health information before entering college. Therefore, conventional school-based sexual health knowledge teaching may be less effective in improving the sexual health of GBMSM college students.

The abovementioned two changes in information seeking have also substantially altered the GBMSM college student’s needs for sexual health support. Rather than access to knowledge and information, currently, the students are longing for a trusted professional that can assist them in examining the reliability of information and to better apply knowledge when they encounter sexual health issues in everyday life, such as psychological distress, condom use, HIV rapid testing, and pre- and post-exposure prevention measures. In the students’ narratives, such personnel, whom they named as a “health manager”, is imagined and desired. For this “health manager”, the qualities of understanding LGBTQ living experiences and providing corresponding emotional affirmations were highlighted by our GBMSM college student participants. This expression echoed what Sun et al. [3] pointed out in their literature review: addressing the psychological and emotional aspects of Chinese MSM could be a direction for tackling their HIV-related health behaviors.

GBMSM college students’ envision of a trusted and thoughtful “health manager” reminds us that services for HIV prevention and sexual health are human interactions that involve people’s emotional connectedness and interpersonal experiences [23]. Due to China’s mainstream sociocultural environment’s repression of sexual and gender minorities, one’s disclosure of minority sexual and gender orientation and sexual behaviors may derive unwanted stigma and discrimination from their living contexts. Most of the students appear to be cautious in terms of seeking support for sexual health concerns in real life, as expressing the concerns may inevitably be associated with exposing their sexualities. Because of anticipated stigma and discrimination, GBMSM college students naturally turn to those with whom they can trust and emotionally connect for sexuality and sexual health, especially during times of crisis. Our findings reveal that students often place their trust and dependence on other members of local LGBTQ communities, particularly the staff of LGBTQ-led CBOs. The trust is fostered by shared sexual and gender orientations, and similar emotional and embodied experiences. Considering this, we suggest that LGBTQ-led CBO can be a suitable choice for the development of a “health manager” profession aimed at addressing HIV prevention and sexual health needs among GBMSM college students in China.

In other countries, promoting HIV prevention measures among young GBMSM is reported as boosted by peer communities and vernacular knowledge dissemination. A study carried out among men who had sex with men and female sex workers in Nigeria indicated that over 90% of the research participants had heard about preexposure prophylaxis for the first time from community channels [24]. In Southeast Asia, peer networks have served as a vital resource for LGBTQ youth (aged 15–24) in terms of not only HIV-related challenges but also sexual and gender identity and mental health issues [25]. Meanwhile, online tools are observed as increasingly involved in community-based initiatives. In the US, community-informed online HIV prevention programs for adolescent sexual-and-gender-minority males were reflected by the users as useful and significantly improved their HIV knowledge [26]. Involving in-person and online LGBTQ communities has been proven to be effective in preventing HIV transmission and improving the sexual health of young GBMSM worldwide, and may also be able to make contributions in China.

Our study also discussed how the emotional aspect of relational contexts affects the behaviors of GBMSM college students in seeking sexual health support. LGBTQ communities have been an essential player in global strategies for promoting HIV prevention and addressing the HIV pandemic [27]. Since the 2000s, community-based organizations have also been supported in China to implement HIV prevention interventions among key populations, including GBMSM [12]. Previous studies have usually portrayed the emergence of communities of sexual and gender minorities as being bonded by shared political agendas and health interests [27,28]. We discover that emotional connectedness and interpersonal attachments substantially glue GBMSM individuals together and affect their choices of seeking information and service. Qualitative and quantitative research can be directed to further explore (1) the factors that contextualize GBMSM college students’ entry and experience in LGBTQ communities and (2) how emotion and affect work in the formation of communities and further alter students’ support-seeking behaviors in terms of sexual health challenges.

Our study has several limitations. First, the project selected only five cities in China to conduct data collection. Although these cities were selected to include diversified samples and narratives of GBMSM college students and stakeholders, our data might not be able to comprehensively represent the living experiences of all Chinese GBMSM college students. Second, the student participants were recruited through online recruitment and snowballing sampling. The methods might cause some selection bias, as respondents of the recruitment posts might be those who were more attentive to their sexual health than other GBMSM college students. Third, the HIV status of the GBMSM college student participants was not addressed in the data collection and analysis, and differences between the health needs of HIV-positive and -negative students were not explored.

## Conclusion

Our study shows that the access of GBMSM college students to information and services for sexual health has undergone changes in the era of expanding online social media platforms and the development of community-based organizations. When compared to traditional school-based education, these community-based channels have become more appealing to GBMSM college students because of their inclusiveness, accessibility and ability to foster emotional connections. Conventional school-based education, on the other hand, is seen as having discriminatory tendencies and lacking of desired level of accessibility and emotional connection for GMSM college students. The findings highlight the strong desire among GBMSM college students for personalized sexual health support, such as a LGBTQ-affirmative “health manager” who can address specific needs of GBMSM college students. Future research on how emotions and interpersonal contexts influence the decisions of GBMSM college students in seeking sexual health support is highly needed.
